# Use of Nebulized Amphotericin B in the Treatment of Allergic Bronchopulmonary Aspergillosis in Cystic Fibrosis

**DOI:** 10.1155/2010/376287

**Published:** 2010-12-23

**Authors:** M. Proesmans, F. Vermeulen, M. Vreys, K. De Boeck

**Affiliations:** Department of Pediatrics, Cystic Fibrosis Reference Center, University Hospitals Leuven, 3000 Leuven, Belgium

## Abstract

*Background*. Systemic steroids and adjunctive antifungal therapy are the cornerstone in treating allergic bronchopulmonary aspergillosis (ABPA) in the context of CF. *Aim*. Evaluate the use of inhaled amphotericin B (iAMB) as antifungal agent in this context. *Methods*. Report of 7 CF patients with recurrent or difficult to treat ABPA and failure to taper systemic corticosteroids treated with AMB deoxycholate (AMB-d) (Fungizone 25 mg 3× a week) or AMB lipid complex (ABLC) (Abelcet 50 mg twice weekly). Successful therapy was defined as steroid withdrawal without ABPA relapse within 12 months. *Results*. Therapy was successful in 6 of 7 patients treated with iAMB. In 5/6, lung function improved. The patient with treatment failure has concomitant MAC lung infection. *Conclusion*. Inhaled AMB may be an alternative to commonly used adjunctive antifungal therapy in the treatment of ABPA. More data are needed on safety and efficacy.

## 1. Introduction

Allergic bronchopulmonary aspergillosis (ABPA) is a hypersensitivity reaction to *Aspergillus *species. In children, it is mostly encountered in the context of cystic fibrosis (CF). 

The reported prevalence of ABPA complicating CF lung disease varies from 6 to 25% depending on the age and the diagnostic criteria used [1–3]. The clinical diagnosis is difficult because symptoms are nonspecific and resemble bacterial CF airway infection. Different diagnostic criteria have been established based on a combination of clinical and radiological signs together with the following biochemical parameters, again none of them being very specific: elevated IgE (>500–1000 IU/l or a 4-fold rise), eosinophilia, specific IgE for *A. fumigatus* or recombinant antigens (or positive skin prick test), and precipitins to *A. fumigatus*. [3]. 

Systemic corticosteroids are still the cornerstone of therapy starting at a dose of 1-2 mg/kg/day for 2-3 weeks [4], but the pace of tapering is highly variable and individualized. Adjunctive antifungal agents like itraconazole (200–400 mg/day for several months) may reduce corticosteroid need. However, few studies involving only a small number of CF patients have been published [3, 5]. 

In our CF center, ABPA is usually treated with a combination of systemic steroids and itraconazole. In patients with frequent ABPA flares or failure of steroid tapering, inhaled amphotericin B (iAMB) is considered as alternative antifungal therapy. Because only scarce data exist [6, 7], we report our experience with this treatment in 7 CF children with a difficult-to-treat ABPA course.

## 2. Methods

Inhaled AMB is considered in case of one or more of the following: (1) insufficient response to initial therapy with corticosteroids and itraconazole (2) frequent ABPA relapse during/after steroid withdrawal despite itraconazole treatment (3) failure to taper systemic steroids (4) intolerance for itraconazole. Data were retrospectively collected on 7 CF patients treated for ABPA with inhaled AMB.

In a first step AMB deoxycholate (AMB-d) (Fungizone Bristol-Meyers Squibb Belgium) was administered as a test dose (20 mg in a concentration of 1 mg/ml; nebulization for 10–15 minutes with a PARI turboboy nebulizer or Aeroneb Go) [8]. If tolerated, therapy was given 3 times a week. In case of intolerance for AMB-d (cough, wheeze, and shortness of breath), AMB-lipid complex (ABLC) was used (Abelcet Wyeth 50 mg in a concentration of 5 mg/ml, 2 ×/week; nebulization time 10–15 minutes). Duration of therapy was individualized (see cases and figures). Choice of the dosage scheme was mainly based on doses used in other indications [9] and the reported long half-life of iAMB in the lung in animal models [10], especially when using a lipid formulation. Because ABLC has a 1/1 molar ratio for the active product versus the phospholipids (see Table 2), the weekly dose was about the double compared to AMB-d. 

Treatment was considered a success if systemic steroids could be stopped without ABPA relapse for at least 12 months after steroid withdrawal. Prednisolone was started at a dose of 1-2 mg/kg/day. After 3 weeks, the patient was seen in clinic, and tapering was started in case of clinical improvement and fall in IgE. If clinically indicated steroids were tapered with 5 mg every 2 weeks. However, final decision for speed of tapering was made by the treating CF physician. Improvement of lung function is not used as an absolute criterion for treatment success in this small patient series because important comorbidities influence lung function evolutions.

Specific IgE for different recombinant *Aspergillus* antigens were measured (f1, f2, f3, and f4) (ImmunoCAP 1000, Phadia; cut-off >0.7 Ua/ml).

**Patient 1 (WS °21-12-2001)**

**Figure 1:**
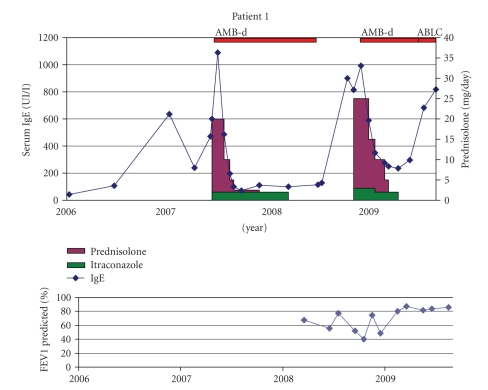


This CF girl had a difficult respiratory course from young age with frequent infectious exacerbations treated with oral and IV antibiotics. ABPA was first diagnosed at age 5 based on a respiratory exacerbation not improving with IV antibiotic treatment combined with a rise in IgE to 1089 kU/l and positive RAST to the recombinant *Aspergillus* antigen f6 (4–6.5 Ua/ml). *Aspergillus* was growing from her sputum. Treatment with oral steroids and itraconazole (Sporanox Janssen-Cilag 1 × 100 mg) was started (Figure 1). Because of persisting atelectasis of the left upper lobe after 2 weeks of oral steroids and itraconazole, inhaled AMB-d was added after which the atelectasis cleared. Sputum remained negative for *Aspergillus*. One year after steroid stop and 8 months after inhaled AMB-d was stopped, ABPA recurred and was successfully treated with the same combination of prednisolone and antifungal therapy (itraconazole and AMB-d). She is now 9 month off steroids, and inhaled AMB-d was changed to ABLC and will be continued until at least 1 year free of ABPA. IgE is however rising again, and a new flare is suspected.

**Patient 2 (BL °13-4-1998)**

From diagnosis, the respiratory course of this girl was unstable with frequent infections and lung infiltrates treated with IV antibiotics. Because of severe gastro-esophageal reflux, a Nissen fundoplication was performed at the age of 5 years. She developed asthmatic symptoms (in the context of familial atopy and asthma) for which she has been treated with inhaled steroids.

**Figure 2:**
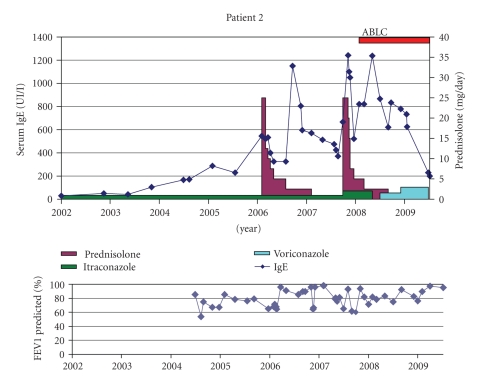


She was diagnosed with a first episode of ABPA based on a respiratory exacerbation not improving with IV antibiotics, IgE rising from 230 kU/l to 500 kU/l, eosinophilia (1200/*μ*l), and a positive RAST for recombinant *Aspergillus* antigen f4 (between 9 and 43 Ua/ml). *Aspergillus* was cultured from sputum. She was treated with oral steroids and itraconazole (Sporanox Janssen-Cilag 1 × 100 mg, later 2 × 100 mg) (Figure 2). Two years later there was a relapse of ABPA. Only slow clinical improvement was seen under oral steroids, and itraconazole was replaced by inhaled ABLC. Because of decreasing lung function and repeated *Scedosporium prolificans* in sputum cultures, voriconazole (Vfend Pfizer 2 × 125 mg increased to 2 × 200 mg/d based on subtherapeutic blood levels) was added. Sputum was initially not clear; however, she finally became free of *Aspergillus* (from march 2009).

Only under combined antifungal therapy, steroids could be stopped and lung function improved. She is now free of oral steroids since 1 year.

**Patient 3 (BW °30-10-1995)**

**Figure 3:**
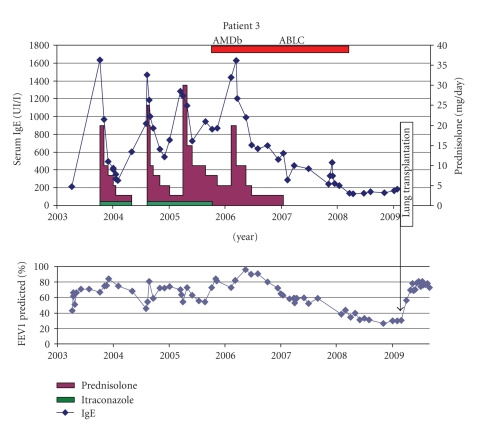


This boy developed chronic lung infection with *B. cepacia* (*multivorans*) from the age of 8. He was diagnosed with ABPA in the same year after a prolonged respiratory exacerbation being not resolved despite repeated antibiotic administration. IgE titer was 1635 kU/l; specific IgE for *Aspergillus* recombinant antigens were positive only for f3 (1 Ua/ml), not for f4 nor f6. He was treated with oral steroids and itraconazole (Sporanox Janssen-Cilag 2 × 100 mg). One year later, he developed an ABPA relapse with prolonged course and difficult steroid tapering (Figure 3). After start of inhaled AMB-d (later switched to ABCL), steroids could be tapered and finally stopped. Inhaled ABCL was stopped after he was free of steroids and ABPA relapse for 1 year. Sputum cultures became negative for *Aspergillus* since start of iAMB.

Despite the resolution of the ABPA, the overall evolution in this CF patient was unfavorable. He developed a chronic lung abscess in the right lung for which a right lower lobe resection was performed at the age of 12. However, chronic *B. cepacia* suppurative infection of the remaining right lung persisted with development of collapse and a functional right lung. He received a lung transplant at the age of 13.5.

**Patient 4 (DK °13-1-1993)**

This girl developed severe obstructive lung disease and bronchiectasis despite intensive treatment and frequent courses of IV antibiotics.

**Figure 4:**
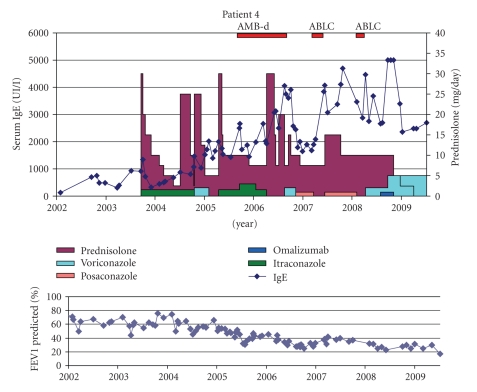


The first episode of ABPA at the age of 10 (Figure 4) was treated with oral steroids and itraconazole (Sporanox Janssen-Cilag 2 × 100 mg). Several attempts to taper the steroids failed. From the age of 11 (2004), Mycobacterium avium-intracellulare (MAC) infection has been documented. Several combination treatments have been given with limited if any success (ethambutol, rifampicin, clarithromycin or azithromycin, IV amikacin, ciprofloxacin or levofloxacin, interferon gamma 1-b). Lung function continued to decline.

Sputum cultures mainly grew *Candida* and *Aspergillus *species despite longstanding treatment with itraconazole. Hair loss was reported. Therefore, treatment with voriconazole (Vfend Pfizer 2 × 120 mg) was started. After 1 month of therapy, slight improvement of lung function and decreased cough were seen. Voriconazole had to be stopped because of severe diarrhoea. Posaconazole (Noxafil Schering-Plough 2 × 400 mg) was given for a certain period but stopped because of reimbursement issue (see Figure 3). Inhaled AMB-d was initiated without clear response and therefore stopped again. ABLC was only given for short periods of time but was disliked by the patient. Sputum initially cleared; however, *Aspergillus* was isolated again after stop of iAMB. Voriconazole was successfully reintroduced at a higher dose (2 × 240 mg daily) based on subtherapeutic blood levels, but IgE remains high (Figure 4) and lung function fails to improve.

**Patient 5 (BP °25-7-90)**

**Figure 5:**
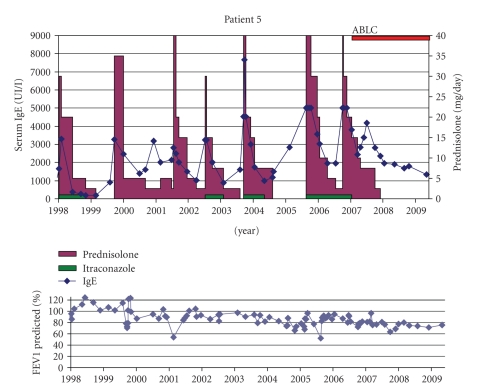


ABPA was diagnosed based on bilateral lung infiltrates not improving under adequate antibiotic therapy combined with raised IgE (3295 kU/l) together with positive RAST and precipitins for *A. fumigates*. She was treated with oral steroids and itraconazole (Sporanox Janssen-Cilag 2 × 200 mg) but had frequent relapses over the years (Figure 5).

Only after starting inhaled ABLC, steroids could be tapered as she has been free of ABPA relapse for almost 2 years although her IgE levels remained high. No more *Aspergillus* was cultured from her sputum.

**Patient 6 (VM °24-7-1989)**

**Figure 6:**
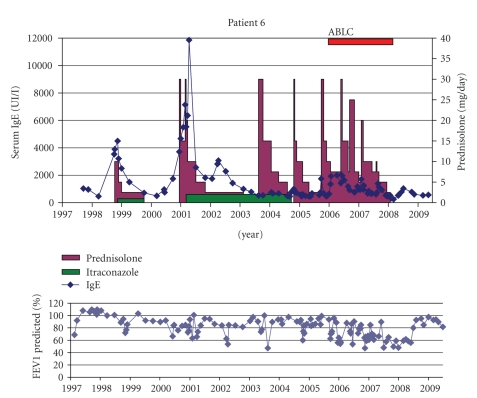


Childhood years were characterized by rather stable respiratory disease, but since adolescence, respiratory exacerbations became frequent. A Nissen fundoplication was performed at the age of 13 because of severe gastro-oesophageal reflux. ABPA was diagnosed at the age of 11 based on a respiratory exacerbation not improving with IV antibiotics and a high IgE of 3890 kU/l. She was treated successfully with oral steroids and itraconazole (Sporanox Janssen-Cilag 2 × 100 mg later 2 × 200 mg) (Figure 6). After a relapse 3 years later, tapering of steroids resulted in frequent ABPA exacerbations. Sputum cultures were intermittently positive for *A. fumigatus*, *C. albicans* and *S. prolificans*. After initial start of inhaled ABLC, compliance was reported as low. Insisting on the importance of the antifungal therapy finally resulted in better adherence resulting not only in successful weaning (more than 2 years off steroids) of therapy but also improved lung function. She remained free of *Aspergillus* in her sputum under treatment with iAMB.

**Patient 7 (ZB °13-10-89)**

Diagnosis of ABPA was first made at the age of 9 based on a respiratory exacerbation with a new lung infiltrate not clearing with antibiotics. IgE was 881 kU/l, with positive specific RAST for *A. fumigatus* as well as positive skin prick test. *Aspergillus* was never grown from her sputa. She was first treated with oral steroids only. At the time of the 3rd ABPA episode, itraconazole (Sporanox Janssen-Cilag 2 × 200 mg) was started, but steroid tapering was difficult.

**Figure 7:**
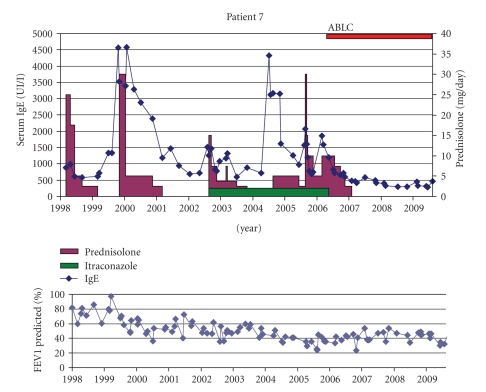


At the age of 17, inhaled ABLC was started because of failure to stop the systemic steroids. Steroids were slowly tapered and successfully stopped after 8 months. She has been free of ABPA since 2.5 years with more stable lung function evolution (Figure 7).

## 3. Discussion

We report the use of inhaled AMB in the treatment of 7 CF children with difficult-to-treat ABPA. For some patients AMB was used in combination with itraconazole or voriconazole. For 5 of the 7 patients treated with inhaled AMB, treatment was considered a success: patients were weaned from systemic steroids without ABPA relapse for at least 12 months. For 2 of these 5 patients, clinical evolution may well be influenced by cotreatment with voriconazole although plasma levels were subtherapeutic. The patient that remained steroid dependent (patient 4) had complex lung disease with MAC infection. In 4 out of 5 successes, lung function improved as well. The patient that had progressive lung function decline, despite successful ABPA treatment, had *B. cepacia* lung infection. 

A consensus guideline on management of ABPA in CF has been published [3]. To reduce the burden of *A. fumigatus* in the respiratory tract it may be worthwhile to treat with antifungal medication [4, 11, 12]. Itraconazole is however lipophilic and may not be well absorbed in CF patients. Use of proton pump inhibitors may further interfere with itraconazole absorption. Other possible drug interactions include CYP3A4 inhibition leading to QT prolongation and decreased methylprednisolone metabolization. Voriconazole, approved for the treatment of invasive aspergillosis, has a better oral bioavailability. It is however expensive, has a high potential for drug interactions, and has been associated with a number of adverse effects [13, 14], and no formal data exist on its absorption in CF. 

In the light of these concerns, use of inhaled medication like AMB is considered. Data on clinical use mainly concern prevention and treatment of invasive *Aspergillus* infections in neutropenic or lung transplant patients [9]. AMB has been the treatment of choice for most invasive fungal infections since its introduction in the 1950s. It has a broad spectrum of activity including *Aspergillus fumigatus*, *Candida albicans,* and *Cryptococcus neoformans*. Lipid formulations have been developed to reduce nephrotoxicity after parental administration while retaining the drug's activity. Main differences between these formulations are listed in Table 2. Clinically they differ mainly in risk for toxicity, dosing, and tissue concentration after parental administration [15]. 

The nebulization of AMB was first studied in a rat model of pulmonary *Aspergillus* infection. Prophylactic treatment before fungal inoculation with either formulation resulted in a significantly prolonged survival for all. Mean concentrations of AMB in the lungs were significantly higher and had a longer half-life with ABLC compared to a similar dose of AMB-d [16]. The liposomal formulation had the longest half-life [10]. 

In lung transplant patients, ABLC and L-AMB are better tolerated than conventional AMB-d. The deoxycholate salt used in conventional AMB-d acts as a detergent, impairing the surfactant function which is a potential toxic effect [17, 18]. In contrast, the liposomal carrier in L-AMB exhibits a pulmonary surfactant-like function.

The clinical use of inhaled AMB has been recently reviewed [9]. Several data indicate that nebulizing AMB is possible and lung deposition can be obtained. Lipid formulations nebulize better than AMB-d. Few reports are found on the use of inhaled AMB in the treatment of ABPA. The use of nebulized AMB-d in the treatment of acute ABPA in a non-CF boy was reported [19]. A dose of 2.5 mg was given 3 times daily, but duration of therapy was not mentioned. Data on 5 CF patients treated weekly with L-AMB was published in abstract form [20]. A dose of 50 mg was given diluted in 8 mL aqua. Duration of nebulization was reported as 150 minutes on average which makes this treatment burdensome. Systemic steroids could be stopped in 4 of the 5 patients. In one patient, ABPA relapsed 7 months after stop of L-AMB. Recently, a short report describes the successful use of nebulized AMB (5 mg two times daily) in 3 CF children with ABPA in combination with inhaled budesonide [7].

Based on pharmacokinetic data, we used a 2- or 3-time per week administration of inhaled AMB. AMB-d was used when tolerated. Based on possible effects on pulmonary surfactant, we try however to avoid longstanding use of AMB-d and switch to ABLC.

With AMB-d, main adverse event was cough and wheeze induced by the nebulization. These side effects were far less prominent with ABLC. None of the patients had to stop the treatment with ABLC because of side effects. Patients however dislike the treatment because of the bad taste.

We acknowledge that this paper is only a retrospective case series. Prospective interventional trails will be needed in order to evaluate the place of inhaled antifungal medication in the treatment of ABPA.

We are aware that recently several case reports have been published on the use of omalizumab in ABPA treatment in CF [21–24]. A prospective randomized trial is currently conducted in Europe. Even if this therapy proves to be efficient for this indication, omalizumab will not be suitable for all CF patients with ABPA, merely because of side effects but also because of high cost and the necessary 2 weekly in-hospital administration.

## 4. Summary

We report on the use of inhaled AMB in the treatment of 7 CF patients with difficult-to-treat ABPA. In 6 of the 7 patients treated, steroids could be stopped and patients remained free of relapse after several years of recurrent ABPA episodes. 

Based on this limited experience, inhaled AMB may be considered as antifungal therapy in the context of ABPA treatment in CF. More data are needed for efficacy and long-term safety. 

##  Conflict of Interests

All authors state that there is no conflict of interests.

## Figures and Tables

**Table 1:** 

	Current age (years)	Age at Λ (months)	Age at first ABPA diagnosis (years)	Sweat Cl (meq/l)	Genotype	Airway bacterial pathogens during ABPA	Indication for inhaled AMB	Other medica issues
1 (f)	7	11	5.5	106	1717 − 1G > A/g.3464_3471dup TCATTGCT and V1198M	SA, SM, AX, Chronic *PA* (age 6)	1	ICS
2( f)	11	4	8	85	F508del/F508del	SA, AX,	1, 2	ICS, Nissen
3(m)	13	2	8	90	F508del/?	*B. cepacia* (age 8)	2, 3	
4 (f)	16	7	10	98	F508del/R553X	Chronic PA (age 10), MAC (age 11)	2, 3, 4	MAC, gastrostomy, DM
5 (f)	19	36	7	120	F508del/F508del	SA, HI, SM	2, 3	DM
6 (f)	20	1 (NS)	11	?	F508del/F508del	Chronic PA (age 16)	2, 3	Nissen
7 (f)	20	At birth	9	95	F508del/F508del	Chronic PA (age 5)	2, 3	MI, DM

**Table 2:** Different formulation of amphotericin B.

Formul- ation	Carrier	Colloidal type	Size (*μ*m)	Molar ratio
AMB-d	Deoxycholate	Micelle	0.035	
L-AMB	PC/DSPG/Chol	Liposome	0.08	2 : 0. 8 : 1 : 0. 4
ABLC	Phospholipids	Lipid ribbon	1.6–11	1 : 1
ABCD	Cholesteryl sulphate	Lipid disk	0.11-0.12	1 : 1

PC: phosphatidylcholine; DSPG: distearoylphosphatidylglycerol; Chol: cholesterol.

## Abbreviations

(i)AMB: (inhaled) Amphotericin B
AMB-d: Amphotericin B deoxycholate
ABLC: Amphotericin B lipid complex
L-AMB: Liposomal amphotericin B
ABCD: Amphotericin B colloidal dispersion
CF: Cystic fibrosis
ABPA: Allergic bronchopulmonary aspergillosis.
